# Tibial tubercule osteotomy during the revision of total knee arthroplasty: The technique of a referral center with 10 years of experience

**DOI:** 10.1051/sicotj/2023016

**Published:** 2023-06-13

**Authors:** Nicolas Cance, Cecile Batailler, Robin Canetti, Elvire Servien, Sébastien Lustig

**Affiliations:** 1 Orthopaedics Surgery and Sports Medicine Department, FIFA Medical Center of Excellence, Croix-Rousse Hospital, Lyon North University Hospital 69004 Lyon France; 2 Univ Lyon, Claude Bernard Lyon 1 University, IFSTTAR, LBMC UMR_T9406 69622 Lyon France; 3 LIBM – EA 7424, Interuniversity Laboratory of Human Movement Science, Université Lyon 1 69008 Lyon France

**Keywords:** Tibial tubercle osteotomy, Revision total knee arthroplasty, Surgical technic, Union, Complications

## Abstract

*Introduction*: The Tibial Tubercle Osteotomy (TTO) technique, by lifting the distal bony attachment of the extensor mechanism, allows efficient knee exposure while preserving soft tissues and tendinous attachments. The surgical technique seems essential to obtain satisfying outcomes with a low rate of specific complications. Several tip sand tricks can be used to improve this procedure during the revision of total knee arthroplasty (RTKA). *Technique*: The osteotomy should be at least: 60 mm in length and 20 mm in width to allow fixation with 2 screws; and 10–15 mm thick to resist to screw compression. The proximal cut of the osteotomy must keep a proximal buttress spur of 10 mm to get primary stability and avoid the tubercle ascension. A smooth end of the TTO distally reduces the risk of a tibial shaft fracture. The strongest fixation is obtained using two bicortical 4.5 mm screws slightly ascendant. *Results*: From January 2010 to September 2020, 135 patients received an RTKA with concomitant TTO and a mean follow-up of 51 ± 26 months [24–121]. The osteotomy was healed in 95% of patients (*n* = 128) with a mean delay of 3.4 ± 2.7 months [1.5–24]. However, there are some specific and significant complications related to the TTO. Twenty complications (15%) related to the TTO were recorded, with 8 (6%) requiring surgery. *Conclusion*: Tibial tubercle osteotomy in RTKA is an efficient procedure to improve knee exposure. To avoid tibial tubercle fracture or non-union, a rigorous surgical technique is primordial with a sufficient length and thickness of the tibial tubercle, a smooth end, a proximal step, a final good bone contact, and a strong fixation.

## Introduction

A step-by-step approach is fundamental to achieving good outcomes during revision total knee arthroplasty (RTKA), starting with adequate exposure which could be complicated by many factors such as stiffness, patella baja, or previous scars [1]. Furthermore, the constraints applied to the extensor mechanism can lead to its rupture and dramatic consequences [2]. Different exposure techniques have been described: quadriceps snip [3, 4], V-Y quadriceps plasty [4], and tibial tubercle osteotomy (TTO) [5–7]. The TTO technique, first described by Dolin [8] and then modified by Whiteside and Ohl [9] showed encouraging results. Lifting the distal bony attachment of the extensor mechanism allows sufficient exposure of the knee while preserving soft tissues and tendinous attachments.

According to the literature, it represents a safe and efficient procedure in a native knee or during TKA, with a high union rate, a poor complication rate, and good clinical results [10, 11]. Several surgical techniques have been described in the literature [9, 12–14]. The surgical technique seems essential to obtain satisfying outcomes with a low rate of specific complications. This technical note aims to describe our surgical technique for tibial tubercle osteotomy during revision total knee arthroplasty, with 10 years of experience in a referral arthroplasty center.

## Materials and methods

From January 2010 to September 2020, 135 patients received an RTKA with concomitant TTO and with minimum two-year follow-up data. The mean follow-up was 51 ± 26 months [24–121].

The main indication for TTO during RTKA is a difficult exposure, commonly due to *patella baja*, stiffness (flexion < 70° or flexion contracture > 20°), or previous TTO. TTO is also performed to remove a well-fixed tibial implant or a cement plug in case of a PJI or to improve patellar tracking. A weakness of the extensor mechanism (previous patellar fracture, patellar tendon reconstruction, etc.) can be a relevant indication of TTO to reduce the constraints on the extensor mechanism. Iterative TTO was not a contraindication. TTO should be avoided when a hinged implant is used. In our practice, we do not use quadriceps snip or VY plasty for an extensive approach. The main indications for RTKA were infection (*n* = 68; 50%).

## Surgical technique

### Step 1: Planning

TTO is usually planned preoperatively, according to the range of motion and patella height. In a few cases, TTO is decided during the surgery when proper exposure is impossible without damaging the extensor mechanism, or if patellar tracking is not satisfying. If the TTO is performed to remove the tibial implant or cement plug, it is necessary to measure on the preoperative radiographs the needed length of the TTO to reach the end of the stem or cement plug.

Equipment required for a tibial tubercle osteotomy includes:

- 3.2 and 4.5 mm drills (or 2.7 and 3.5 mm drills for 3.5 screws),
- 4.5 mm screws (or 3.5 screws),
- Eventually, wire cerclages, if the fixation by screws is not possible,
- Chisels,
- Oscillating saw, classically using a thin and large blade such as the one used for patellar cut.

### Step 2: Standard approach

**Figure 1:**
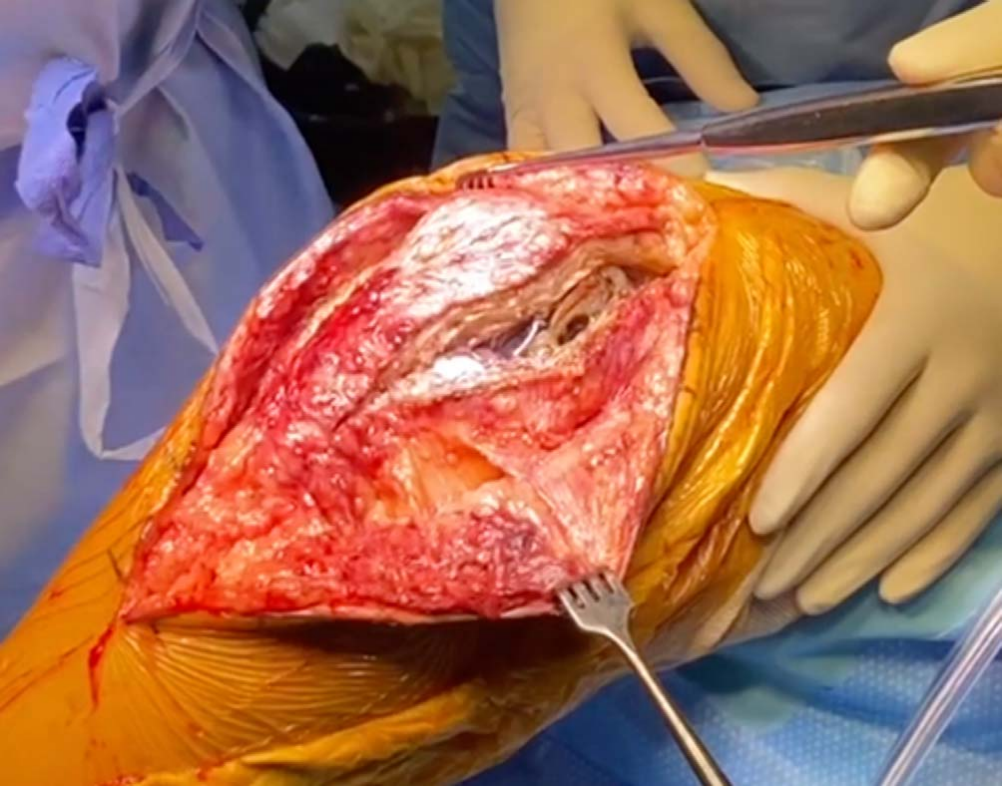
Parapatellar arthrotomy and tibial tubercle exposition.

Patients are in a supine position, with the knee bend at 90° of flexion. The skin incision is standard, according to the lateral or medial approach and previous scar, extended 5–10 cm distally to allow tibial tubercle exposure (Figure 1). We usually performed an anteromedial approach with a medial parapatellar arthrotomy (subvastus) for straight and varus knees, and an anterolateral approach using a modified Keblish approach for valgus knees. Medial or lateral approaches can be used with TTO without significant differences in exposure. The approach can also be a combined medial and lateral subvastus arthrotomy, with the TTO everted proximally, but with an increased risk for patellar vascularity.

### Step 3: Sizing

The osteotomy should be at least:

- 60 mm length and 20 mm width to allow fixation with 2 screws.
- 10–15 mm thick to resist screw compression and have cancellous bone contact.

**Figure 2:**
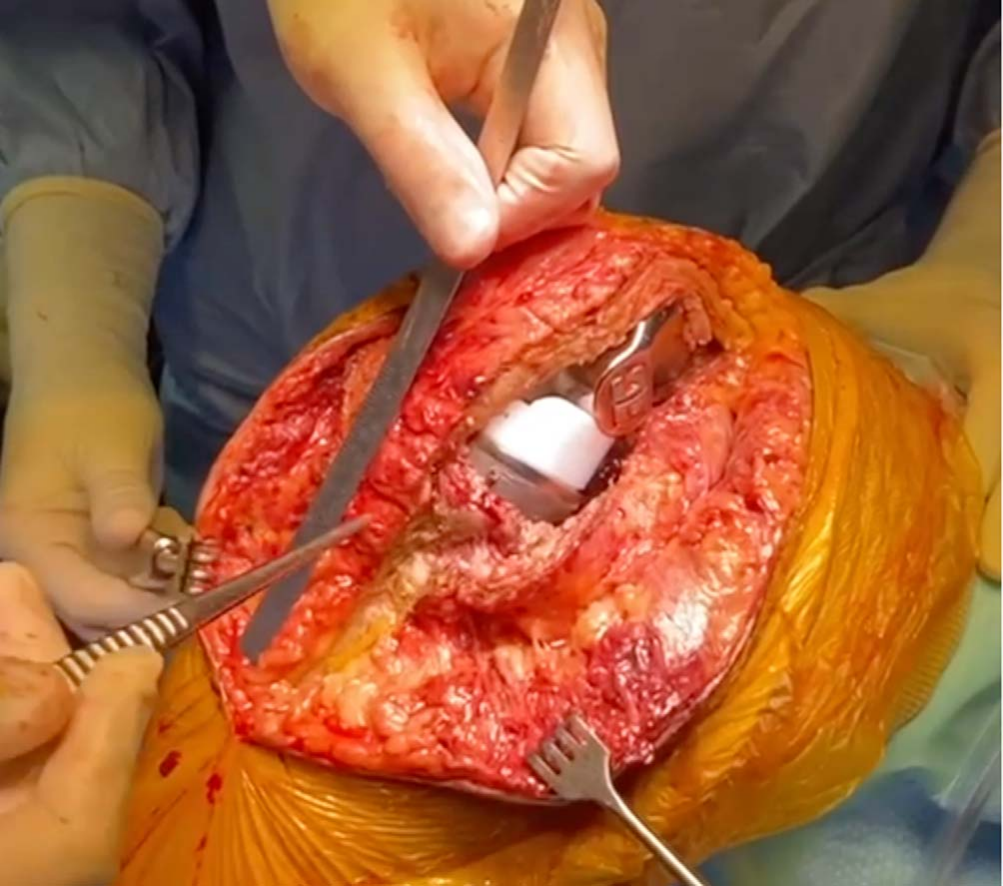
Bone block sizing.

**Figure 3:**
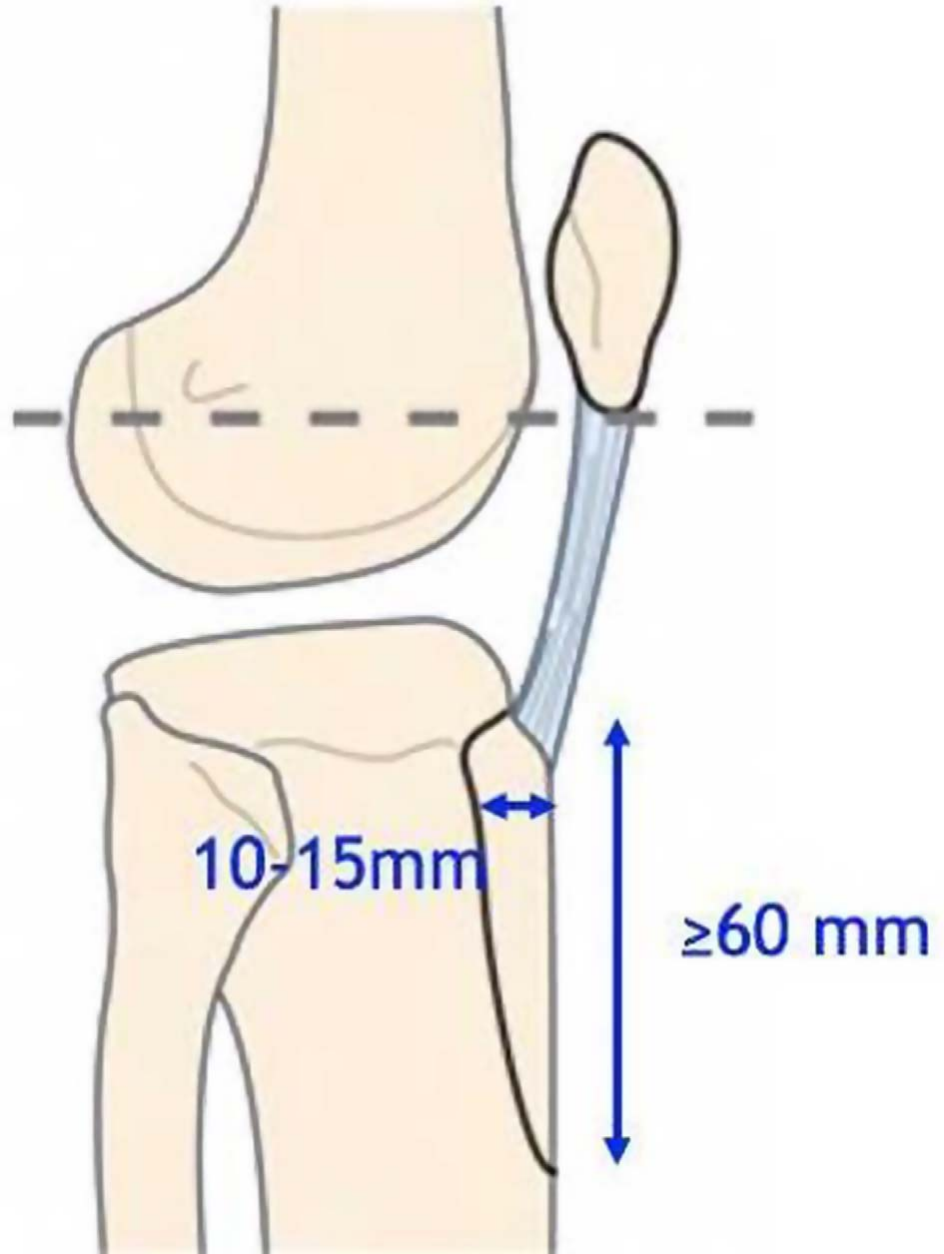
Bone block sizing.

Bone sizing is one of the essential parts of this surgical technique (Figures 2 and 3). A smaller bone block could lead to its fracture during fixation or knee flexion. A bigger bone block could lead to a tibial diaphyseal fracture or direct contact with the tibial stem or cement.

Before bone cutting, the tibial tubercle is drilled to prepare the position of the screws, using a 3.2 mm drill bicortical, then a 4.5 mm drill for the anterior cortex (Video 1).

### Step 3: Osteotomy

**Figure 4:**
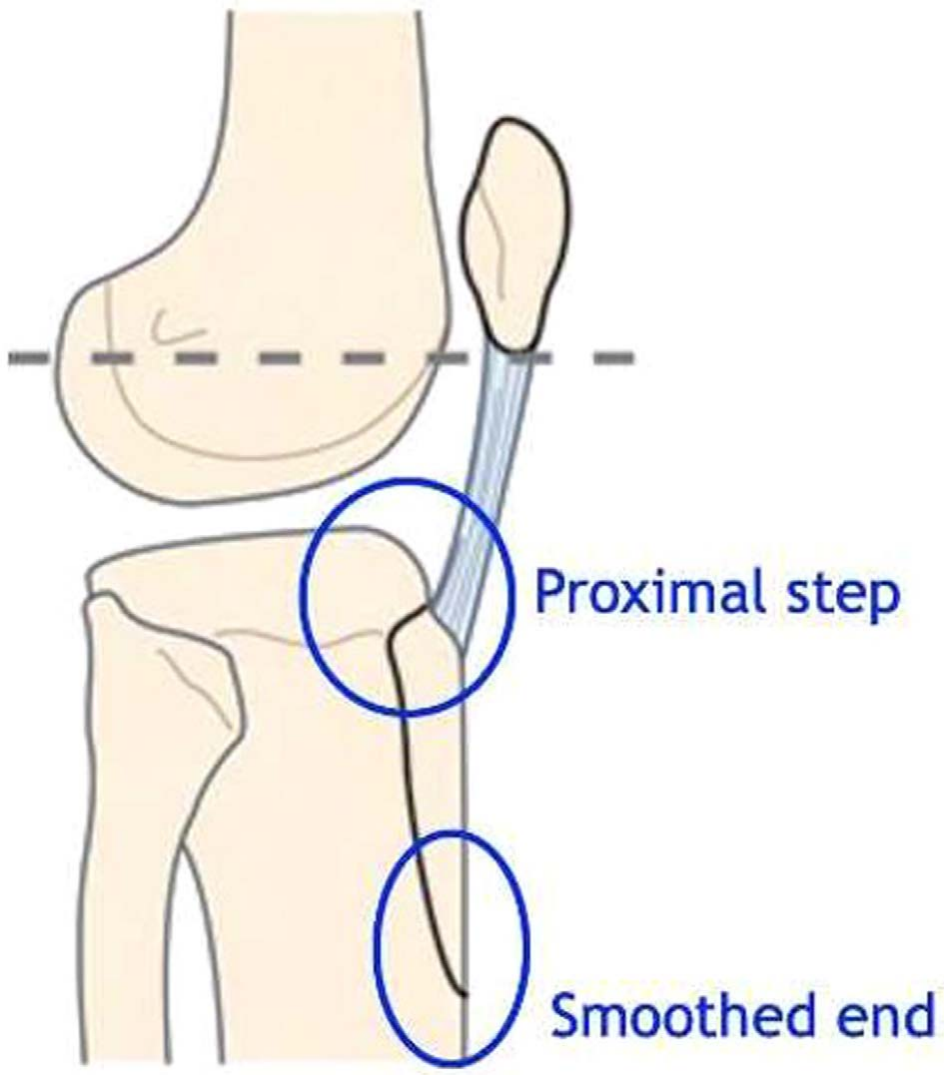
Tibial tubercle osteotomy with proximal buttress spur and smoothed end.

**Figure 5:**
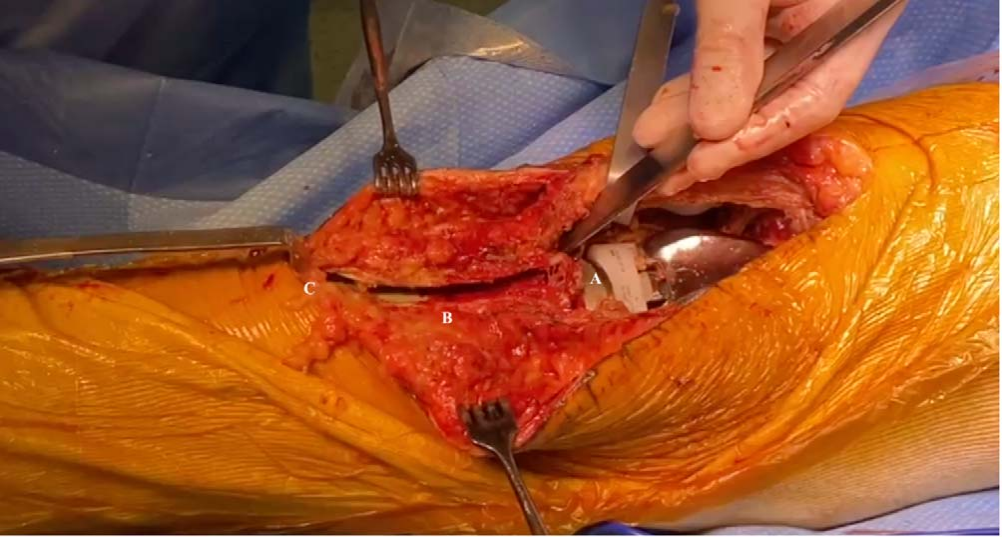
Tibial tubercle osteotomy during RTKA. (A) A proximal bone bridge was preserved using chisels to avoid migration of the tibial tubercle. (B) The osteotomy commenced medially using a saw cut parallel to the tibia shaft. The bone block size is at least 60 mm long and 10 mm thick. (C) A smooth chamfered end distally is used to reduce the risk of a tibial shaft fracture.

**Figure 6:**
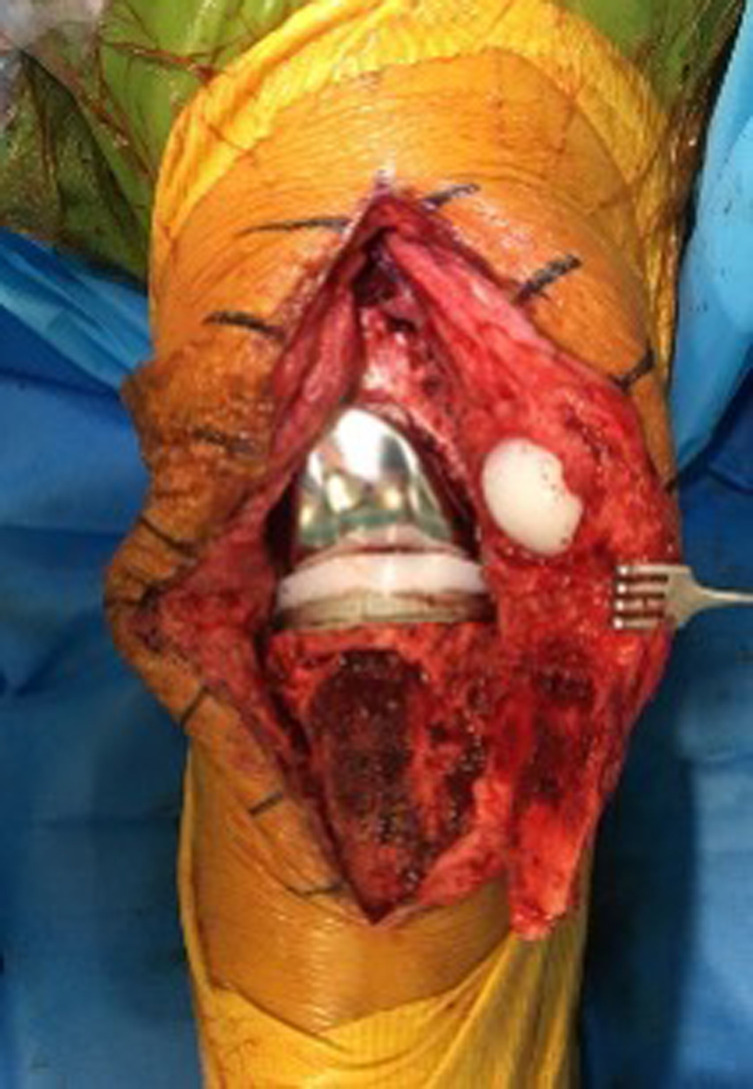
Panoramic view after tibial tubercle osteotomy, put on the lateral side.

The osteotomy is performed using an oscillating saw starting proximally. According to the scar, the osteotomy starts medially or laterally using a saw cut parallel to the tibia shaft. Both sides are wholly sawn through. The lateral muscular sleeve is retained as far as possible. The proximal cut of the osteotomy must not go through the articular surface but keep a proximal buttress spur of 10 mm to get primary stability and avoid the tubercle ascension (Figures 4 and 5, Video 2). This proximal buttress spur is obtained by a vertical cut with a chisel. A chamfered end distally reduces the risk of a tibial shaft fracture (Figure 6, Video 3).

### Step 4: Arthroplasty revision

Implants are removed using chisels. If necessary, tibial implant removal can be facilitated by hitting the stem using intramedullary access created by the osteotomy. Once revision trial implants are placed, flexion and patellar position are checked with the tibial tubercle maintained in proper position with an awl.

Essential care must be taken:

1. Protect the anterior tibial plateau cortex during tibial preparation to avoid spur or sagittal tibial fracture. A tibial augment or sleeve can be used to improve the metaphyseal fixation.
2. Tibial stem should bypass the distal end of TTO to avoid diaphyseal fracture.
3. Be careful with the tibial component rotation. The medial part of the tibial tubercle is frequently used as a landmark for the tibial plateau rotational position but cannot be used after the osteotomy. Care must be taken to avoid tibial malrotation. Tibial crest, foot position, or dynamic positioning between the femur and polyethylene could be used.
4. Protect osteotomy bone contact. The cement between the osteotomy and the tibial shaft should be removed to maximize bone union.

### Step 5: Tubercle positioning and fixation

After the definitive implants are cemented, the tibial tubercle is positioned anatomically in extension. It is then maintained with an awl or fingers, and the patellar position is checked while flexion is applied (Video 4). It is possible to adjust the tibial tubercle position to improve patellar tracking. A sub-quadricipital release is done to increase extensor mechanism mobility for stiff knees. Biomechanical studies showed the strongest fixation is obtained using two bicortical 4.5 mm screws slightly ascendant (Video 5). Screws are measured to be bicortical (measurement +2 mm). Final compression is obtained with a manual screwdriver, taking care not to break the tubercle. Washers are not used in usual practice to reach a satisfying compression of the osteotomy. Osteotomy fixation is then checked by applying at least 90° flexion.

When a metaphyseal cone or sleeve compromises the fixation by 4.5 mm screws, we use three 3.5 mm bi-cortical screws. Screws are used wherever possible. We use wire cerclages when the bone is weak (thickness < 10 mm) or when screws can’t be used due to a bulky metaphyseal cone or sleeve. To medialize the tibial tubercle, the distal screw is placed first, without completely tightening the screw. This allows the tibial tubercle to be rotated around the screw and translates the proximal tibial tubercle medially before the proximal screw is placed. The distal screw is then tightened.

**Figure 7:**
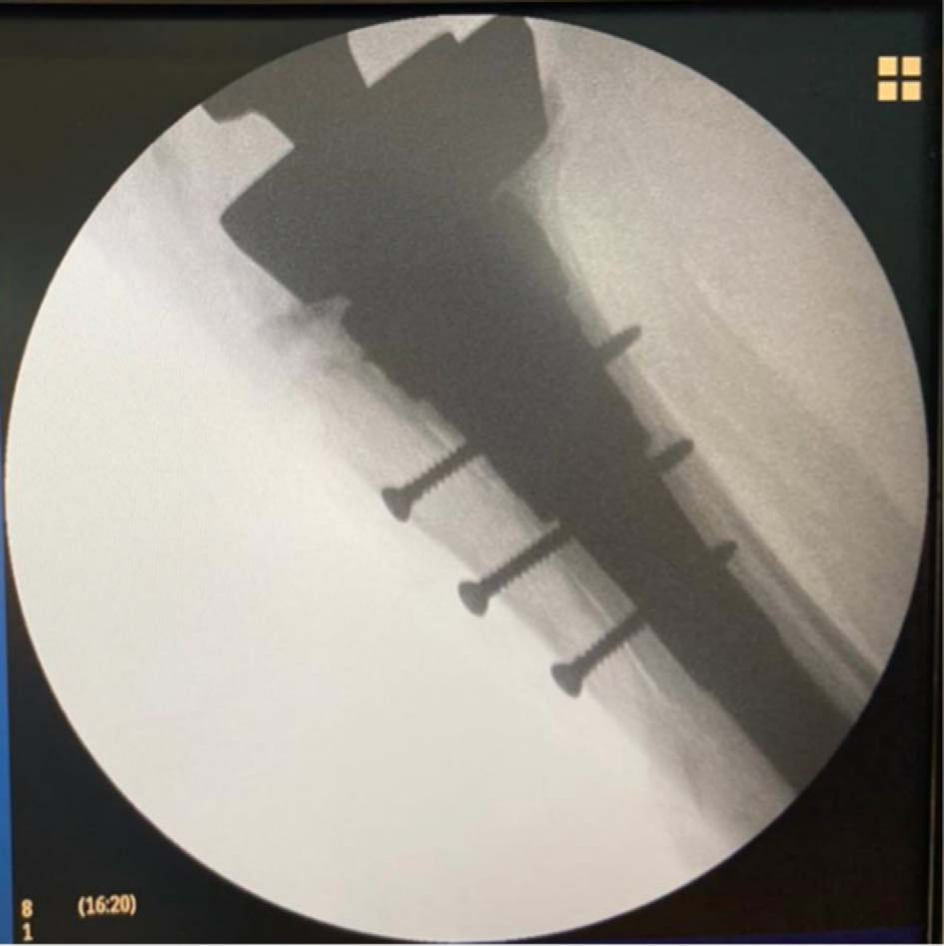
X-ray control.

The final control is done using fluoroscopy (Figure 7).

### Step 6: Postoperative rehabilitation protocol

**Figure 8:**
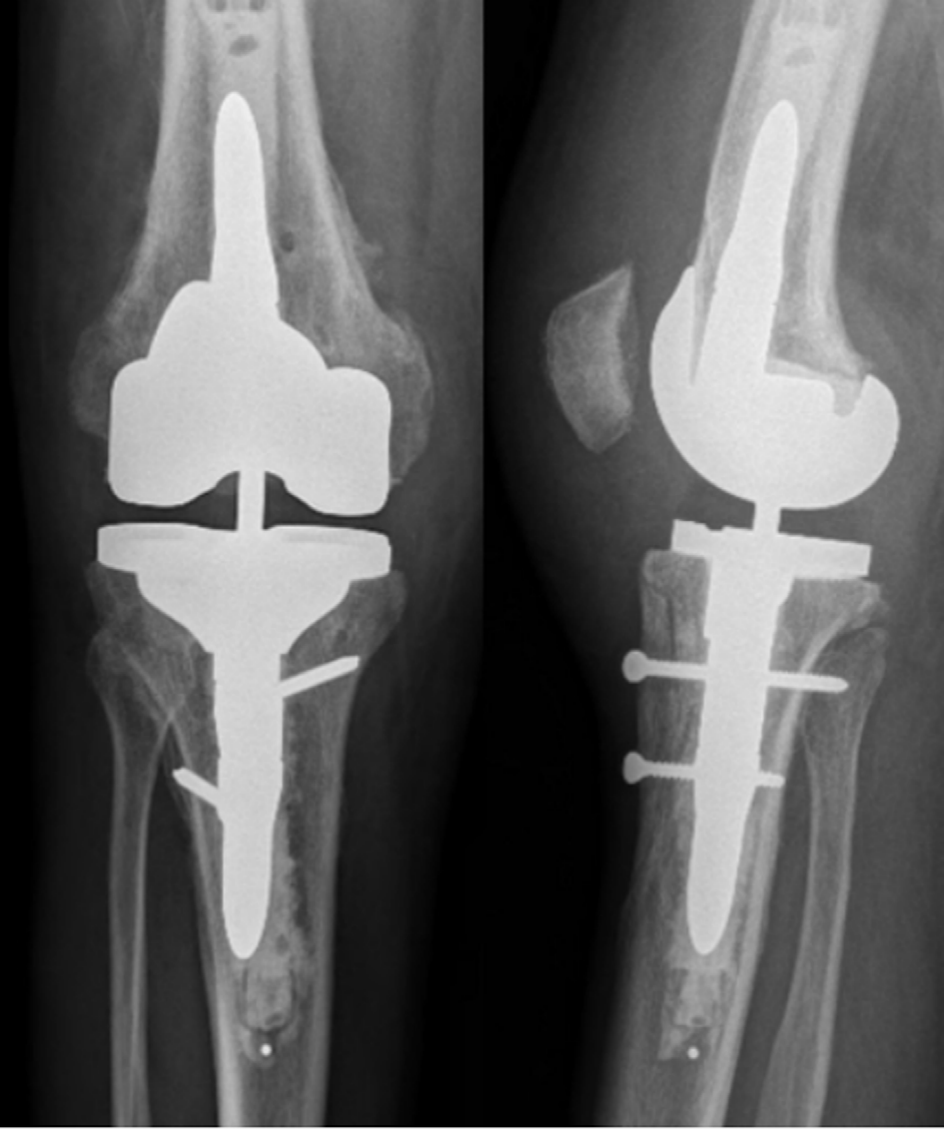
X-ray control at 3 months, confirmed bone union.

An immediate postoperative extension brace is applied to the knee. Our rehabilitation protocol allows immediate full weight bearing with the leg locked in full extension with a brace. Flexion while non-weight-bearing was limited to 95° for 6 weeks. Maximal flexion was allowed 6 weeks postoperatively, after a clinical and radiological examination (Figure 8).

All TTOs used the described surgical technique. The osteotomy was round at the distal part for 123/135 patients (91%) with a mean of 9.1 ± 1.7 cm [5.9–14.4] long and 1.2 ± 2.7 cm [0.6–2.0] thick. The TTO fragment is reduced back to its anatomic position for 109/135 patients (81%).

## Results

The osteotomy was healed in 95% of patients (*n* = 128) with a mean delay of 3.4 ± 2.7 months [1.5–24]. However, there are some specific and significant complications related to the TTO. Twenty complications (15%) related to the TTO were recorded, with 8 (6%) requiring surgery.

Fracture displacement of the TTO (9/20; 45%) and aseptic non-union (5/20; 20%) were the most common. Fracture displacements were treated with reduction osteosynthesis; and. non-union with open-reduction and bone graft.

Functional outcomes found a full flexion improvement of 12°, an improved KSS Knee, and a Function score of 31 and 33 points respectively. There was no post-operative extension lag.

## Conclusion

TTO in RTKA is an efficient procedure to improve knee exposure. To avoid tibial tubercle fracture or non-union, a rigorous surgical technique is primordial with a sufficient length and thickness of the tibial tubercle, a smooth end, a proximal step, a final good bone contact, and a strong fixation. The rehabilitation protocol must be standardized with an immediate full weight bearing with an extension brace.

## Supplementary material

The supplementary material of this article is available at https://www.sicot-j.org/10.1051/sicotj/2023016/olm. *Video 1*. Bone block sizing and pre-drilling tibial tubercle. *Video 2*. Tibial tubercle osteotomy. *Video 3*. Panoramic view after tibial tubercle osteotomy. *Video 4*. Positioning of the bone block and checking of the patellar tracking. *Video 5*. Screws fixation of the tibial tubercle.
